# Determinants of Community Mental Health Service Utilization Among Psychiatric Outpatients at a Tertiary Medical Institution: Applying Andersen’s Behavioral Model

**DOI:** 10.3390/healthcare13090996

**Published:** 2025-04-25

**Authors:** Min Hee Jung, Sung Hee Shin

**Affiliations:** 1 Major in Psychiatric Mental Health Nurse Practitioner Program, Graduate School of Nursing, Kyung Hee University, Seoul 02447, Republic of Korea; jmh3578@khu.ac.kr; 2 College of Nursing Science, Kyung Hee University, Seoul 02447, Republic of Korea

**Keywords:** mental disorder, health literacy, stigma, depression, stress

## Abstract

**Objectives:** This study examined factors influencing community mental health service utilization and identified strategies to improve accessibility for psychiatric outpatients receiving care at the Department of Psychiatry. **Methods:** This cross-sectional descriptive survey utilized a structured questionnaire to collect data from 136 patients receiving outpatient psychiatric care at a tertiary medical institution in Seoul, Korea. Logistic regression analysis was performed using SPSS version 26.0 for Windows. **Results:** Logistic regression analysis identified significant factors influencing the utilization of community mental health services: being a man (OR = 3.33), duration of illness (OR = 2.31), recognition of service institutions (OR = 39.09), internalized stigma (OR = 4.90), and stress (OR = 3.14). **Conclusions:** To encourage the utilization of community mental health services by patients with mental illness, psychiatric nurses should increase the level of understanding and information about community-based mental health support. Additionally, details on community mental health services should be provided from the earliest stages of illness in a variety of gender-specific ways during discharge education, and patients with high levels of internalized stigma and stress should be encouraged to have an insight of their illness and to take an active role in their recovery.

## 1. Introduction

The lifetime prevalence of mental disorders in South Korea is 27.8%, indicating that approximately one-third of the population will experience mental health issues such as alcohol or nicotine use disorders, anxiety disorders, or depressive disorders at least once in their lifetime [1]. Additionally, in most countries, including South Korea, the number of individuals with mental disorders and the costs associated with mental health issues has been steadily increasing [2]. In particular, schizophrenia and mood disorders, which are major mental illnesses, are challenging to treat in a short period because of the nature of the illness. Many cases progress to a chronic course after repeated improvements and relapses, causing a significant social and economic burden for patients, their families, and the country [3]. In response, the government provides various community mental health services to prevent relapses among individuals with chronic mental disorders and assist in their social reintegration and recovery [4]. According to Fleury et al. [5], 46.5% of individuals in Canada who had been diagnosed with a mental health issue within the past year accessed mental health services. In Australia, by contrast, 34.9% of individuals diagnosed with a mental disorder used such services over a 12-month period [6]. However, it has been reported that only 12.1% of individuals diagnosed with mental disorders utilize community mental health services [1]. Specifically, the characteristic tendency of mental disorders to become chronic, along with the increasing number of individuals with mental illnesses and associated healthcare costs, shows that the utilization rate of mental health services in South Korea remains low. Consequently, it is imperative to examine the factors that impact the utilization of community-based mental health services to promote access among individuals with chronic mental disorders.

Since the enactment of the Mental Health Act in 1995, mental health services in Korea have shifted to community-centered services, highlighting the significance of minimizing the hospitalization of patients with mental illness and providing treatment in communities where patients reside [7]. Community mental health services are crucial for the continuous management, rehabilitation, and reintegration of individuals with mental disorders into their communities [8].

In a study by Joe et al. [9], based on the medical law-based classification, patients admitted to psychiatry departments were divided into general hospitals, hospitals, and clinics, and the results of analyzing the most frequent diseases among patients admitted to psychiatry departments under health insurance by hospital type were as follows: depression, schizophrenia, and bipolar disorder were diagnosed in that order in psychiatry departments of general hospitals, while schizophrenia, alcohol use disorder, and pervasive developmental disorder were diagnosed in that order in clinics. Based on this, it can be assumed that many patients with the above-mentioned diagnoses are also among outpatients in psychiatry departments of general hospitals. Schizophrenia and mood disorders are difficult to treat in a short period of time, and they often progress to a chronic course with repeated worsening and improvement [3]. Therefore, identifying factors that affect participation in community mental health support services among outpatients in psychiatry departments of general hospitals can help in the relapse and recovery of the subjects’ illnesses. In the treatment of people affected by mental illness, insight is the most fundamental motivating factor in utilizing services. Therefore, it is necessary to determine which other factors, aside from insight, determine access to community mental health care among outpatients receiving services from psychiatric departments.

Accordingly, several studies have been carried out on the utilization of community mental health services by individuals with mental disorders. When examining studies on the use of community mental health services, Kim and Kahng [10] investigated factors affecting the use of mental health services by using the experience of using mental health services as a dependent variable, and found that age, education level, duration of illness, and the service needs of mentally ill people who were not diagnosed with schizophrenia were factors affecting the use of community mental health services. Cheon and Choi [11] mentioned social support as a factor affecting the willingness to use services and service use behavior in an analysis of the use of mental health services targeting community residents and stated that it helps solve or reduce mental health problems through a social support system in times of depression or stress. Fleury et al. [5] confirmed that mental illness and emotional problems were the main factors affecting the use of mental health services for people with and without mental illness. At the same time, as a limitation of the study, Kim and Kahng [10] mentioned that the level of service use may vary depending on the level of awareness of the service, that social discrimination or stigma may act as a hindrance to the use of the service, and that additional variables related to these should be considered. Cheon and Choi [11] stated that the social support system is one of the key factors affecting the use of mental health services, and that it is necessary to divide it into formal and informal support systems and confirm the differences in mental health behaviors according to each type. In the study by Graham et al. [12], secondary data were utilized, which limited the ability to measure certain variables that the researchers intended to examine, particularly those affecting mental health service utilization. A notable limitation of the study lies in its inability to fully consider a wide range of factors that may act as barriers to, or directly impact, service utilization. Furthermore, reliance on secondary data restricted the ability to capture specific variables directly associated with mental health service use.

Factors that have been identified in previous studies to date regarding community mental health services include internalized stigma [13], social support [14], mental health literacy [15], depression [11], and stress [16]. Internalized stigma describes the procedure through which people with mental illness internalize stereotypes, prejudices, and discrimination associated with mental illness through social cognition, leading them to feel incompetent and worthless, and consequently avoid utilizing mental health services. This is recognized as an obstacle to obtaining mental health care for individuals with mental disorders [13]. Social support is fundamental to the causes and relapse of mental illness in patients and has a direct impact on treatment and rehabilitation [14]. In a study by Lian, Wallace, and Fullilove [14], spouses, family, and friends were essential factors in providing support for accessing mental health services. Mental health literacy refers to using related resources to respond appropriately to situations as well as the knowledge and understanding of mental health issues [15]. Mackenzie and Pankratz [15] reported that mental health literacy is essential in increasing the use of mental health services. Conversely, depression can be defined as a state in which it is difficult to maintain one’s usual lifestyle owing to a depressed mood, decreased motivation or pleasure, decreased concentration, decreased appetite, and sleep problems. Moreover, individuals also experience difficulties in interpersonal relationships and social activities [17]. In previous studies, depression has been reported as a factor affecting mental health service use [11]. Additionally, for patients with mental illness, stress is closely related to their surroundings and changes in their lifestyle. Therefore, they are susceptible to stress and overreact in crises, and illness occurs or relapses even with minor stress [16]. Yorgason, Linville, and Zitzman [16] reported that elevated stress levels were linked to a higher utilization of mental health services.

Andersen’s behavioral model of health service utilization (henceforth referred to as Andersen’s behavioral model) has been employed to validate the utilization of health services based on individual characteristics [10,18]. This model focuses on the predisposing, enabling, and need factors. Predisposing factors are personal tendencies that determine whether to use a service before using it. Enabling factors promote or hinder the use of individual or community services. Further, need factors are health needs that are subjectively perceived or objectively evaluated by the individual and directly affect service use [18,19]. Therefore, this study aimed to explore the factors affecting the utilization of community mental health care among patients with mental illness who were confirmed to have the will and motivation to receive psychiatric treatment by regularly visiting the outpatient psychiatry department of a tertiary medical institution and applying Andersen’s behavioral model. This study selected the following predisposing factors, enabling factors, and need factors according to previous studies using Anderson’s behavioral model.

Kim and Kahng [10] identified age, level of education, and the length of illness as significant predisposing variables affecting the utilization of mental health services, while the diagnostic status of schizophrenia emerged as a critical determinant within the domain of need factors. In the study by Oh [20], mental health literacy was found to be a factor influencing the intention to use mental health services. Reinhold, Magaard, and Brütt [21] verified internalized stigma as an enabling factor. Cheon and Choi [11] demonstrated that the social support system functions as a primary enabling factor, whereas levels of depression and stress significantly contribute to the perceived need for engagement in community-based mental health services. Based on previous studies, sex, age, marital status, educational level, and duration of illness were identified as predisposing factors; internalized stigma, social support, mental health literacy, and income as enabling factors; and depression, stress, and diagnosis as need factors.

Following this, the study model was formulated by establishing variables that affect the use of mental health services. This was to confirm their influence on community mental health services utilization, thereby ultimately seeking ways to promote community mental health services utilization by patients with chronic mental illness.

## 2. Materials and Methods

### 2.1. Study Design and Participants

The descriptive cross-sectional study was designed to investigate the factors that influence community mental health services use among patients with mental illness who are receiving outpatient services in the psychiatric department of a tertiary medical institution. The study surveyed convenience sampling subjects who met the selection criteria among the mentally ill patients using the outpatient service of the psychiatry department of a tertiary medical institution in Seoul.

### 2.2. Participants

The selection criteria for the participants included the following: First, individuals aged 18 years or more who had been diagnosed with schizophrenia spectrum disorder, bipolar disorder, or depressive disorder by a psychiatrist. These conditions often progress to a chronic course with repeated relapses and exacerbations once they occur, making continuous management and rehabilitation necessary to prevent this [3]. Second, those who were receiving outpatient treatment because their acute symptoms were stable and were judged not to be dangerous to themselves or others. Third, those who did not have any organic disorders or neurological diseases. Fourth, those who were capable of communicating, reading, and understanding the questionnaire. The target number of participants was determined using the G-power 3.1.9.7 application. A previous study on predictive factors for mental health service use [11] was used as a reference to conduct a logistic regression analysis. An odds ratio of 2.27 was obtained, with a significance level of 0.05 and a power of 0.95, and the minimum sample size was 132. Accordingly, accounting for a 10% dropout rate, a survey was conducted with 150 individuals. Data from 136 participants, excluding dishonest responses, were part of the final evaluation.

### 2.3. Measurements

#### 2.3.1. Community Mental Health Utilization

The study comprised 112 questions, including 15 on general characteristics and community mental health service utilization, 29 on internalized stigma, 12 on social support, 35 on mental health literacy, 20 on depression, and 1 on stress. This was measured using a structured questionnaire, and each tool was used after receiving approval from the original author and translator.

The experience of using community mental health services was measured by checking whether there was any experience of using psychosocial rehabilitation facilities (community mental health centers, day hospitals, and day rehabilitation programs), vocational rehabilitation, group homes, and other facilities based on the Mental Health Act. If there was even one use, it was measured as 1; otherwise, it was measured as 0.

#### 2.3.2. Internalized Stigma

Internalized stigma was measured using the Korean Version of the International Stigma of Mental Illness Scale (ISMI) developed by Ritsher and Phelan [22] for patients with mental illnesses and adapted and standardized for Koreans by Hwang et al. [23]. This measure is made up of 29 items overall, categorized into five sub-factors: alienation (six items), stereotype endorsement (seven items), discrimination experience (five items), social withdrawal (six items), and stigma resistance (five items). This is a self-administered tool using the Likert scale, where scores range from 1 point (Strongly disagree) to 4 points (Strongly agree). For negative items, reverse scoring was applied, and the scores varied from 29 to 116, with higher scores correlating with a higher degree of internalized stigma. The original tool demonstrated a reliability of Cronbach’s α = 0.90 [22]. According to Hwang et al. [23], Cronbach’s α was 0.91, while in the present study, it was 0.93.

#### 2.3.3. Social Support

Social support was assessed with the Multidimensional Scale of Perceived Social Support (MSPSS) created by Gregory, Nancy, Sara, and Gordon [24] and revised and supplemented by Park [25] for patients with mental illness. This tool comprises 12 items across three factors: family (four items), friends (four items), and significant others (four items). Significant others referred to professionals at community mental health centers, psychiatric rehabilitation facilities, or medical staff at psychiatric hospitals, excluding family or friends. The scale consists of five levels, ranging from 1 (Strongly disagree) to 5 (Strongly agree), with total scores ranging from 12 to 60, where a higher score reflects greater social support. When the tool was developed, Cronbach’s α was 0.91. According to Park’s study [25], Cronbach’s α ranged from 0.80 to 0.85, while in the current study, it was 0.90.

#### 2.3.4. Mental Health Literacy

Mental health literacy was assessed with the Mental Health Literacy Scale (MHLS) developed by O’Connor and Casey [26] and translated and revised by Cho and Choi [27]. This tool consists of 35 items, and the sub-factors are as follows: the ability to recognize disorders (eight items), knowledge of where to seek information (four items), knowledge of risk factors and causes (two items), knowledge of self-treatment (two items), knowledge of professional help available (three items), and attitudes that promote recognition or appropriate help-seeking behavior (16 items). Items 1 to 15 were evaluated using a four-point Likert scale, while items 16 to 35 were assessed using a five-point Likert scale. The reverse items were reverse-converted, and the score range was 35–160; a higher score indicates a greater level of mental health literacy. The original tool [26] demonstrated a reliability of Cronbach’s α = 0.87. According to Cho and Choi [27], Cronbach’s α was 0.80, while in the present study, Cronbach’s α = 0.82.

#### 2.3.5. Depression

Depression was evaluated using the Center for Epidemiologic Studies Depression Scale (CES-D), developed by Radloff [28] and translated by Cho and Kim [29]. The CES-D is an instrument designed to assess the occurrence of depression symptoms in the previous week and consists of 20 items. Each item is rated on a four-point Likert scale, with responses ranging from 0 (none of the time) to 3 (all of the time), yielding scores from 0 to 60. The reverse items were processed using reverse coding. As the score increases, there is a greater level of depression. The original tool [28] demonstrated a Cronbach’s α of 0.91, while Cho and Kim [29] reported a Cronbach’s α of 0.91; in the present analysis, Cronbach’s α was 0.92.

#### 2.3.6. Stress

Stress was measured using a numerical rating scale (NRS). The evaluation guidelines ranged from 1 point (not at all) to 5 points (very severe). Further, the participants were instructed to select the number that represented their stress level. A higher rating signified a higher degree of stress experienced by the participants.

### 2.4. Procedures

The data collection period was from 27 September to 26 October 2023, and before data collection, the Institutional Review Board (IRB) of the Seoul National University Hospital approved the research (H-2308-137-1495). The specific data collection method involved visiting the outpatient psychiatry department of a tertiary medical institution beforehand, providing an overview of the study’s purpose and methods to two psychiatrists, and seeking their cooperation. Subsequently, the researcher visited the outpatient psychiatry department on the participants’ scheduled appointment dates. With the assistance of outpatient nurses, it was confirmed that the participants met the selection criteria. The aim of the research, the guarantee of anonymity, and participants’ rights were then explained. Informed written consent was acquired from individuals who consented to participate in the research. Data were obtained via a structured questionnaire. When filling out the questionnaire, the researcher directly explained any parts the participants had difficulty understanding to help them understand the questions. The average time to complete the questionnaire was 15–20 min, and the researcher checked and collected the completed questionnaires immediately.

For ethical considerations, the aims and methodology of the research were comprehensively explained to the participants before the survey, and written consent was obtained before the research was conducted if they had agreed to participate. The researcher assured the participants that the questionnaire would be conducted anonymously and that the gathered information would be anonymized and used exclusively for study objectives, with no other intended purpose. Participants were informed that they could withdraw from the study without any disadvantages. They were also informed that the collected data would be stored in a locked cabinet that only the researcher could access. Further, they would be stored for three years after the end of the research and then destroyed. A token of appreciation was provided to the participants in the study.

### 2.5. Statistical Analysis

The gathered data were examined using SPSS software version 26.0 for Windows. First, a descriptive statistical analysis was conducted to identify the general characteristics of the study subjects and characteristics associated with the use of community mental health services. Second, a chi-square test (χ^2^ test) was performed to determine whether community mental health services were utilized based on the general characteristics of the research subjects and the factors associated with the use of these services. Third, a descriptive statistical analysis was performed to examine the internalized stigma, social support, mental health literacy, depression, and stress levels of the participants. Fourth, Pearson’s correlation analysis was performed to assess multicollinearity between the independent variables, and logistic regression analysis was carried out to examine the determinants of community mental health service usage.

## 3. Results

### 3.1. General Characteristics of Participants and Characteristics Related to Community Mental Health Services Utilization

The research subjects’ general characteristics and the characteristics associated with the utilization of community mental health services are shown in <Table 1>. A total of 72 men (52.9%) and 64 women (47.1%) participated in the study. The average age was 37.4 years (±11.4), with 43 participants (31.6%) in their twenties, 42 (30.9%) in their thirties, and 51 (37.5%) aged 40 and above. Marital status included 113 individuals (83.1%) who were single single, 14 (10.3%) married, and 9 (6.6%) other. The educational level of the participants included 51 (37.5%) who had completed high school or lower, 77 (56.6%) who were college graduates, and eight (5.9%) who had completed graduate-level education or higher. The monthly family income was less than KRW 2 million for 41 individuals (30.1%), KRW 2–3 million for 33 (24.3%), KRW 3–4 million for 22 (16.2%), and KRW 4 million or more for 40 (29.4%). The diagnoses included schizophrenia spectrum disorder in 79 patients (58.1%), bipolar disorder in 45 (33.1%), and depressive disorder in 18 (13.2%), with multiple responses. The average duration of illness was 11.18 (±9.23) years, with fewer than 5 years for 41 participants (30.1%), 5–10 years for 29 (21.3%), 10–20 years for 41 (30.1%), and 20 years or more for 25 (18.4%).

Regarding community mental health services, 91 individuals (66.9%) were aware, and 45 (33.1%) were unaware. For subjects who were aware of community mental health services, the recognition routes in the multiple responses were mass media/advertisements for 20 people (22.0%), acquaintances for 35 people (38.5%), mental health experts such as staff and teachers at related organizations for 55 people (60.4%), related studies and education for 6 people (6.6%), direct questions/exploration for 13 people (14.3%), and others for 3 people (3.3%). Specifically, most individuals became aware of them through mental health experts, such as employees of related organizations or teachers, followed by acquaintances and mass media or advertisements. Community mental health services were used by 45 individuals (33.1%). The results of a survey with multiple responses regarding the community mental health services they had used were as follows: community mental health centers for 30 individuals (22.1%), mental health rehabilitation facilities for 25 (18.4%), and vocational rehabilitation facilities for 3 (2.2%). The reasons for not using community mental health services were as follows: 33 individuals (36.3%) did not know about the service, 25 (27.5%) did not need it, 10 (11.0%) had no one to recommend it, 8 (8.8%) did not want the service, 6 (6.6%) were afraid of being known as having a mental illness, 5 (5.5%) had difficulty going owing to psychiatric symptoms or disabilities, 3 (3.3%) did not like the physical environment, and 1 (1.1%) was for economic reasons.

### 3.2. Differences in Community Mental Health Services Utilization Based on the Participants’ General Characteristics and Characteristics Related to the Use of Mental Health Service

The differences in the utilization of community mental health services based on the participants’ general characteristics are presented in <Table 2>. Community mental health services use was judged by whether the participants had ever used community mental health services—45 participants (33.1%) were found to have used community mental health services.

We verified the differences in community mental health services utilization based on the general characteristics. Significant differences were observed in sex (χ^2^ = 5.09, *p* = 0.024), education level (χ^2^ = 9.36, *p* = 0.009), schizophrenia spectrum disorder diagnosis (χ^2^ = 10.71, *p* = 0.001), bipolar disorder diagnosis (χ^2^ = 5.20, *p* = 0.023), and duration of illness (χ^2^ = 20.42, *p* < 0.001). Regarding sex, the rate of community mental health service utilization was relatively higher for men than for women. Concerning education level, the community mental health service utilization rate was relatively higher for those who had completed high school or less. Those diagnosed with schizophrenia spectrum disorder exhibited a relatively greater rate of community mental health service utilization compared to those without. Furthermore, those without bipolar disorder demonstrated a relatively greater rate of community mental health service utilization compared to those with bipolar disorder. The longer the illness duration, the higher the community mental health service utilization rate.

### 3.3. Differences in Community Mental Health Service Utilization Rates Depending on Whether Community Mental Health Services Are Recognized

The differences in the utilization of community mental health services according to participants’ awareness of these services are presented in <Table 3>. There was a significant difference in the use of community mental health services depending on whether they were recognized (χ^2^ = 28.94, *p*< 0.001). There was a significant difference depending on whether the recognition was made through acquaintances (χ^2^ = 5.10, *p* = 0.024) or mental health professionals such as staff and teachers at related organizations (χ^2^ = 19.20, *p* < 0.001). More specifically, individuals who were aware of community mental health services exhibited a higher level of usage. When individuals became aware of these services through acquaintances, mental health professionals such as staff at related organizations, and teachers, the level of community mental health service utilization was greater.

### 3.4. Internalized Stigma, Social Support, Mental Health Literacy, Depression, Stress, and Level of Use of Community Mental Health Services

The participants’ internalized stigma, social support, mental health literacy, depression, stress, and the utilization of community mental health services are presented in <Table 4>. Internalized stigma was evaluated on a scale ranging from 1 to 4, with the mean score being 2.20 (±0.49), and social support was evaluated on a scale ranging from 1 to 5, with the mean score being 3.25 (±0.84). Mental health literacy was evaluated on a scale ranging from 1 to 5, with the mean score being 3.15 (±0.35), and depression was evaluated on a scale ranging from 0 to 3, with the mean score being 0.97 (±0.64). Stress was evaluated on a scale ranging from 1 to 5, with the mean score being 3.29 (±0.94).

### 3.5. Factors Affecting the Use of Community Mental Health Services

Before identifying the factors influencing the use of community mental health services, a Pearson’s correlation analysis was conducted to check for multicollinearity among the independent variables. Internalized stigma was negatively correlated with social support (r = −0.36, *p* < 0.001) and mental health literacy (r = −0.20, *p* < 0.023) and positively correlated with depression (r = 0.64, *p* < 0.001) and stress (r = 0.45, *p* < 0.001). Social support showed a positive correlation with mental health literacy (r = 0.21, *p* = 0.012) and a negative correlation with depression (r = −0.19, *p* = 0.027). Mental health literacy showed a negative correlation with depression (r = −0.20, *p* = 0.022). Depression showed a positive correlation with stress (r = 0.63, *p* < 0.001). Thus, the r values between independent variables ranged from −0.19 to 0.64, confirming the absence of multicollinearity.

The outcomes of the logistic regression analysis performed to identify the factors influencing the utilization of community mental health services by individuals with mental health disorders are presented in <Table 5>. The classification accuracy of the logistic regression model was approximately 83.1%, and Nagelkerke R^2^ was 0.60, showing an explanatory power of approximately 60%. The Hosmer–Lemeshow chi-square value was 3.96, and the significance probability was 0.861, which was insignificant. Therefore, this logistic regression model can be considered appropriate. As a result of the regression coefficient significance test, sex and duration of illness showed significant results for general characteristics. Furthermore, the likelihood of utilizing community mental health services was significantly greater in men than in women (odds ratio [OR] = 3.33, *p* = 0.025). Additionally, the longer the duration of illness, the higher the likelihood of using community mental health services (OR = 2.31, *p* = 0.009). Further, whether they were aware of the mental health service organization showed significant results. When they were aware of the mental health service organization, the likelihood of using community mental health services was significantly higher (OR = 39.09, *p* = 0.010). Among the variables in the form of scales, internalized stigma showed significant results: the higher the internalized stigma, the greater the possibility of using community mental health services (OR = 4.90, *p* = 0.049). The higher the stress level, the greater the chance of utilizing community mental health services (OR = 3.14, *p* = 0.016). The findings confirmed that the possibility of using community mental health services was significantly higher among men, those with longer illness duration, those who were aware of service organizations, those with higher levels of internalized stigma, and those with higher levels of stress.

## 4. Discussion

This research was carried out to determine the factors influencing the utilization of community mental health services among patients with mental illness using outpatient medical services at the Department of Psychiatry of a tertiary medical institution in Seoul, to examine the factors influencing the utilization of community mental health services by patients with mental illness, and to seek ways to encourage the utilization of community mental health services. For this purpose, Andersen’s behavioral model was used, which was designed to explain individuals’ use of health services and is employed to identify factors that influence health service utilization based on personal and social structural dimensions. In addition, it is composed of predisposing factors, enabling factors, and need factors in determining health services, making it logically clear. Therefore, it can be considered useful in identifying the factors that influence community mental health services utilization [10,11,18,19]. Andersen’s Behavioral Model provides a theoretically robust and practical framework for understanding and predicting the factors that influence the use of health services [18,30]. One of the model’s key strengths lies in its structured categorization of influencing factors into predisposing, enabling, and need components. This structure allows for a comprehensive analysis that considers not only individual-level determinants but also social and environmental factors [30,31].

In this study, awareness of community mental health services was most often obtained through mental health professionals, such as staff or teachers at related institutions providing mental health services, and the most common reason for not using these services was “not knowing”. In other words, the service utilization rate was high when community mental health services were recognized and when recognition was made through acquaintances, staff, or teachers who provided mental health services. Despite reports indicating the quantitative expansion of community mental health services in Korea and the active conduct of mental health-related campaigns and promotions [1,7], individuals with mental illness are still not aware of these services. Additionally, they continue to underutilize them. This suggests a need for a new approach to promoting and encouraging the use of mental health resources. Specifically, a system needs to be established in which mental health professionals working at facilities providing mental health services can directly provide information or service resource education to patients with mental illness.

Additionally, in this study, the response that they did not use mental health services, “they did not need the services”, was the second highest after “because they did not know”. Considering the research results, which indicate that the utilization of community mental health services by patients with mental illness contributes positively to their treatment and rehabilitation, mental health professionals need to actively provide opportunities and education for patients with mental illness to raise their perspectives on mental health services so that they can recognize that community mental health services are helpful and necessary.

The Korean government has identified the low utilization rates of mental health services as being primarily due to limited access to reliable mental health information, inaccurate information, and prejudices surrounding mental health. To address this issue, the government aims to develop and provide accurate, trustworthy mental health information to improve public awareness. By utilizing both domestic and international information channels and expert groups, the government intends to create high-quality content, which will be disseminated through the National Mental Health Information Portal. Additionally, the government plans to organize and provide mental health service availability and utilization methods from the perspective of service users, considering different life stages and situations. Efforts include distributing promotional leaflets and enhancing information accessibility through links on institutional websites. In order to promote positive perceptions of mental health services, the government is developing a mental health awareness measurement system to assess understanding of mental health disorders, as well as levels of stigma and acceptability. Furthermore, to avoid the perpetuation of misconceptions about mental disorders, the government is providing medical information and supporting the production of psychiatric dramas, which will allow both the public and families to indirectly experience the daily lives of individuals with mental health disorders [32].

Early psychosis is a broader concept than first-episode psychosis because it includes the prodromal period, which is the state before the onset of apparent psychotic symptoms and is considered to occur within a maximum of five years from the onset of apparent symptoms [33]. Given that 41% of the participants in this study had been diagnosed with the disease for less than five years, it can be said that there are many subjects with early psychosis. The institution that conducted this study operates the Seoul Youth Clinic and provides tests and treatment for early psychosis as well as treatment programs. Accordingly, it can be estimated that 41% of the study subjects had a disease duration of less than 5 years.

Early intervention is crucial because it can reduce psychiatric symptoms and prevent relapse and chronicity. In addition, early intervention can improve functional aspects, especially social and occupational. A study by Harrison et al. [34] confirmed that participants’ functional levels improved owing to implementing early intervention services, including psychosocial education, stress management, and social skills training. Some reports providing community-based mental health services to individuals diagnosed with early onset psychosis help prevent relapse and promote recovery [35]. Therefore, it is necessary to actively disseminate information about patients with mental illness corresponding to early onset psychosis using community mental health services, as this will help prevent the progression of patients with mental illness to chronic illness.

Furthermore, the findings of this study revealed that men, those with a longer duration of mental illness, those who were aware of the mental health rehabilitation facility, those with greater levels of internalized stigma, and those with elevated levels of stress were more inclined to utilize community mental health services. Numerous studies have demonstrated that women utilize mental health services at higher rates than men [11,19]. Socially, men are more likely to believe that asking for help from others is a sign of weakness, so they are less willing to seek help when they are in a difficult situation [35]. This could be associated with the lower utilization of mental health services based on gender. Gender differences in the use of medical services for mental health reasons can be explained by cultural values and expectations associated with a specific gender or the particular roles supported by men and women. It has also been suggested that women tend to confide in friends and family more often. Weak social relationships were directly associated with an increased use of mental health services [5,12]. In cultures where masculinity is emphasized for men, mental illness may lead them to seek help from formal institutions rather than confiding in close personal connections.

However, in this study, men were over three times as likely to use community mental health services as women. This corresponds to a recent mental health intervention program for men [36], which reported notably increased rates of mental health service utilization following the program. This may be related to the specificity of the program for early schizophrenia at the recruitment site of this study. Therefore, there is a role for nurses or mental health professionals to increase gender-specific publicity and campaigns to encourage more patients to utilize community mental health services. Moreover, within this research, patients with a longer illness duration were more likely to use community mental health services, which supports the previous findings of Kim and Kahng [10].

This study showed that the more significant the degree of internalized stigma, the greater the frequency of mental health service utilization. This result contrasts with that of a previous study [13], which stated that internalized stigma may act as a limiting factor in using mental health services. However, individuals exhibiting greater degrees of internalized stigma tend to hold more judgmental views and insight about mental illness but also show more flexibility in their attitudes and greater efforts to cope with the illness [37,38]. From this perspective, internalized stigma is negative and can be seen as a coping mechanism to overcome one’s situation by using mental health services. Internalized stigma is a challenging factor in the lives of people with mental illness, and the process of overcoming it can be stressful. However, in such situations, it is possible to objectify oneself and gain insight into the illness. Therefore, education through community mental health services is needed to overcome internalized stigma, reduce stress, and enable people to cope well with illness and society. As a psychiatric nurse, you can encourage patients with mental illness to verbalize the anxiety or negative emotions they may experience due to internalized stigma and provide emotional support for the stress caused by stigma. You can help patients overcome stigma by providing accurate information and education about mental illness so that they can have a recoverable perspective on mental illness and look at their situation more objectively. You can explain the justifications for the necessity of mental health services and their advantages in overcoming stigma and play a role in encouraging the utilization of mental health services.

In addition, this research demonstrated that the greater the levels of stress, the more probable the use of community mental health services. This finding aligns with studies [11,16], which suggest that as stress levels increase, the likelihood of utilizing mental health services also rises. This means that the mentally and socially vulnerable group may have a relatively higher demand for mental health services. Therefore, it is necessary to identify the mentally vulnerable group, assess their needs, and devise an approach so that they can easily access and use mental health services. As a role of a psychiatric nurse, it is important to evaluate the stress level of patients with mental disorder, educate them on stress management methods, and provide information on using community mental health services. It is necessary to approach stress management differently depending on age, daily life, and level of social activity. It is also possible to educate the subjects on techniques for managing stress on their own or explain how to use mobile apps. In addition, information on places where community mental health services are implementing stress management programs can be provided and participation encouraged.

Fruthermore, the rate of utilization of community mental health services was high when patients with mental illnesses were recognized by acquaintances, staff members of related organizations, or teachers. According to Jun et al. [4], patients with mental illness receive mental health services from their families or acquaintances. Despite frequent hospitalizations and discharges, medical institutions rarely provide information on community mental rehabilitation service resources. Since mental illness progresses to chronic illness through relapse and worsens throughout treatment [3], appropriate discharge education for mentally ill patients and their families discharged from hospitals may be meaningful. This will increase the patient’s understanding of the disease and improve the subject’s ability to reintegrate into society and take care of themselves by utilizing appropriate community mental health service resources. In a previous study, mental health professionals and acquaintances were found to be the most common when the route of use of mental rehabilitation facilities for patients with mental illnesses was confirmed. Mental health professionals and acquaintances were the highest [4].

In this research, it was verified that mentally ill patients receive information from mental experts have an increased utilization rate of mental health services. It is necessary to educate the subjects so that they can recognize their mental health status and seek help from mental experts when they need support and so that they can use community mental health services. Psychiatric nurses can be closest to the subjects and provide continuous interest and care to build trust in the patients, and through this, they can lead the subjects to make positive choices about their treatment or recovery. Therefore, if psychiatric nurses provide effective education to the subjects based on an understanding of the necessity of community mental health services and related knowledge, they can contribute significantly to enhancing the utilization of community mental health services by the mentally ill. Therefore, when considering the discharge of patients with mental illness, having psychiatric nurses provide discharge education to patients and their families plays a vital role regarding treatment, rehabilitation, and recovery and is a convenient approach in terms of expanding the utilization of community mental health services. As nurses, it is urgent to disseminate information pertaining to community mental health services, as well as to develop systematic education and educational materials for discharge nursing education for patients with mental illnesses.

This study collected data from patients visiting the outpatient psychiatry department of a single tertiary medical institution in Seoul, South Korea; therefore, one should be careful when extending the results to all patients with chronic mental illness, as there are limitations in the generalizability of the findings. Repeated investigations are required to broaden the age range of the participants, the level of medical institution, and the region. In addition, to increase the utilization rate of mental health services within the community for those with mental disorders, it is necessary to confirm various variables other than the utilization factors in this study. Although this study identified key factors associated with the use of mental health facilities, its cross-sectional design limits the ability to infer causal relationships. Future research employing a longitudinal approach would help distinguish between temporary barriers and persistent structural or psychological obstacles to accessing mental health services. Although the present study was conducted based on the minimum sample size calculated using G-power, which was sufficient to ensure statistical significance, there are limitations in terms of the reliability of odds ratio (OR) estimations and external validity. However, given the accessibility of clinical settings and the absence of ethically sensitive data, future studies should consider expanding the sample size to enhance the reliability and generalizability of the findings.

Nonetheless, this study used Andersen’s behavioral model to identify the influence of internalized stigma, social support, mental health literacy, depression, and stress on the use of community mental health services. This study is noteworthy as it reveals factors that impact community mental health services by utilizing the Andersen’s behavioral model in a situation where research on the use of community mental health services targeting outpatients in the psychiatry departments of general hospitals that use mental health services at medical institutions is insufficient. The significance of this study is that it provides a basis for the need for a discharge education program to enhance the utilization of community mental health services by patients with mental illnesses, as the utilization rate of community mental health services proves to be high when community mental health services are recognized.

## 5. Conclusions

The research attempted to identify the extent of community mental health service usage and awareness among patients with mental illnesses utilizing outpatient medical services in the psychiatric department of a tertiary medical institution. Additionally, it aims to propose a strategy for increasing access to community mental health services by examining how internalized stigma, social support, mental health literacy, depression, and stress impact the use of these services. The research findings confirmed that male sex, illness duration, awareness of mental health service organizations, internalized stigma, and stress were variables that influenced the use of community mental health services.

The nursing implications of this study are outlined as follows. First, in terms of nursing practice, psychiatric nurses serve as key personnel in promoting the utilization of community-based mental health services among individuals with mental disorders, playing a central role in ensuring continuity of care and rehabilitation following discharge. Nurses must actively intervene during discharge education by equipping patients with adequate knowledge and understanding of available community mental health services, thereby facilitating their ability to recognize and access appropriate services. To achieve this, psychiatric nurses should conduct comprehensive assessments of the patient’s mental health status and individual needs and provide detailed information regarding suitable community-based mental health institutions and services accordingly. Furthermore, nurses are expected to deliver patient-centered education that is tailored to the patient’s level of understanding, addressing the purposes, content, and procedures of various mental health services. In addition, it is essential for nurses to create a supportive environment in which patients can openly express concerns or fears related to service utilization. By actively listening and responding empathetically, nurses can help patients identify and engage with services that best align with their personal needs, thus promoting psychological stability and successful reintegration into the community. Such attentive and individualized approaches contribute significantly to the recovery process and enhancement of the quality of life for individuals with mental illness. Second, from a nursing policy perspective, there is a clear need for education regarding community mental health services. In particular, it is important to provide education on the types, characteristics, and access methods of services offered by various rehabilitation and mental health service institutions within the community. Actively promoting such information and knowledge to service users could facilitate their utilization of community mental health services.

According to the research findings, we provide the following: First, as the present study was conducted by surveying mentally ill patients visiting the outpatient psychiatry department of a tertiary medical institution in Seoul, a repeat study is needed to expand the target population by considering the age of mentally ill patients, hospital size, region, and environment to generalize the results. Second, to foster the utilization of community mental health services by patients with mental illness, it is necessary to develop educational materials that provide information on the types and content of services for accessing community mental health services in discharge nursing education. Finally, the response rate for the reason why people with mental disorders did not seek community mental health services was high because they did not need them. This can hinder the treatment and rehabilitation of individuals with mental disorders and is associated with the underuse of community mental health services. Therefore, continuous campaigns and active promotional activities are required to raise awareness regarding this issue.

## Figures and Tables

**Table 1:** General characteristics and characteristics related to the use of community mental health services (N = 136).

Variables	Categories	*n* (%)
Sex	Men	72 (52.9)
	Women	64 (47.1)
Age (year)	20–<30	43 (31.6)
	30–<40	42 (30.9)
	≥40	51 (37.5)
Marital state	Not married	113 (83.1)
	Married	14 (10.3)
	Other	9 (6.6)
Education	≤High school	51 (37.5)
	College	77 (56.6)
	≥Master	8 (5.9)
Monthly household income	<2000	41 (30.1)
(KRW 1000)	2000–<3000	33 (24.3)
	3000–<4000	22 (16.2)
	≥4000	40 (29.4)
Diagnosis *	Schizophrenia spectrum disorder *	79 (58.1)
	Bipolar disorder *	45 (33.1)
	Depressive disorder *	18 (13.2)
Duration of illness (year)	<5	41 (30.1)
	5–<10	29 (21.3)
	10–<20	41 (30.1)
	≥20	25 (18.4)
Awareness of mental health service	Recognition	91 (66.9)
	Non-recognition	45 (33.1)
Pathways to awareness of mental health services	Mass media or advertising	20 (22.0)
(n = 91) *	Family or friends	35 (38.5)
	Mental health professional	55 (60.4)
	Related studies and education	6 (6.6)
	Direct questions and exploration	13 (14.3)
	Other	3 (3.3)
Utilization of community mental health services	Yes	45 (33.1)
	No	91 (66.9)
Services with prior experience *	Community mental health centers	30 (22.1)
	Mental health rehabilitation facilities	25 (18.4)
	Vocational rehabilitation facilities	3 (2.2)
Reasons for not using the service (n = 91)	Afraid of being identified as having a mental disorder	6 (6.6)
	Owing to a lack of knowledge about the service	33 (36.3)
	Owing to dissatisfaction with the physical environment	3 (3.3)
	Financial reasons	1 (1.1)
	Owing to the lack of encouragement from others	10 (11.0)
	Owing to it not being the service I desire	8 (8.8)
	Owing to the lack of need for the service	25 (27.5)
	Owing to the difficulty of going because of mental health symptoms or disabilities	5 (5.5)

* Multiple response.

**Table 2:** Differences in the use of community mental health services based on the general characteristics of the participants (N = 136).

Variables	Categories	n (%)	Utilization of Community Mental Health Services n (%)		χ^2^	*p*
			Yes 45 (33.1)	No 91 (66.9)		
Sex	Men	72 (52.9)	30 (41.7)	42 (58.3)	5.09	0.024
	Women	64 (47.1)	15 (23.4)	49 (76.6)		
Age (year)	20–<30	43 (31.6)	13 (30.2)	30 (69.8)	1.41	0.494
	30–<40	42 (30.9)	12 (28.6)	30 (71.4)		
	≥40	51 (37.5)	20 (39.2)	31 (60.8)		
Marital state	Not married	113 (83.1)	39 (34.5)	74 (65.5)	0.96	0.618
	Married	14 (10.3)	3 (21.4)	11 (78.6)		
	Other	9 (6.6)	3 (33.3)	6 (66.7)		
Education	≤High school	51 (37.5)	25 (49.0)	26 (51.0)	9.36	0.009
	College	77 (56.6)	18 (23.4)	59 (76.6)		
	≥Master	8 (5.9)	2 (25.0)	6 (75.0)		
Monthly household income	<2000	41 (30.1)	15 (36.6)	26 (63.4)	7.00	0.072
(KRW 1000)	2000–<3000	33 (24.3)	15 (45.5)	18 (54.5)		
	3000–<4000	22 (16.2)	8 (36.4)	14 (63.6)		
	≥4000	40 (29.4)	7 (17.5)	33 (82.5)		
Schizophrenia spectrum *	Yes	79 (58.1)	35 (44.3)	44 (55.7)	10.71	0.001
	No		10 (17.5)	47 (82.5)		
Bipolar disorder *	Yes	45 (33.1)	9 (20.0)	36 (80.0)	5.20	0.023
	No		36 (39.6)	55 (60.4)		
Depressive disorder *	Yes	18 (13.2)	5 (27.8)	13 (72.2)	0.26	0.607
	No		40 (33.9)	78 (66.1)		
Duration of illness (year)	<5	41 (30.1)	3 (7.3)	38 (92.7)	20.42	<0.001
	5–<10	29 (21.3)	10 (34.5)	19 (65.5)		
	10–<20	41 (30.1)	18 (43.9)	23 (56.1)		
	≥20	25 (18.4)	14 (56.0)	11 (44.0)		

* Multiple response.

**Table 3:** Differences in service utilization based on recognition pathways and awareness (N = 136).

Variables	Categories	n (%)	Utilization of Community Mental Health Services n (%)		χ^2^	*p*
			Yes 45 (33.1)	No 91 (66.9)		
Awareness of mental health services	Recognition	91 (66.9)	44 (48.4)	47 (51.6)	28.94	<0.001
	Non-recognition	45 (33.1)	1 (2.2)	44 (97.8)		
Pathways to awareness of mental health services:						
Mass media or advertising *	Yes	20 (22.0)	8 (40.0)	12 (60.0)	0.51	0.477
	No		37 (31.9)	79 (68.1)		
Family or friends *	Yes	35 (38.5)	17 (48.6)	18 (51.4)	5.10	0.024
	No		28 (27.7)	73 (72.3)		
A mental health professional *	Yes	6 (6.6)	30 (54.5)	25 (45.5)	19.20	<0.001
	No		15 (18.5)	66 (81.5)		
Related studies and education *	Yes	55 (60.4)	3 (50.0)	3 (50.0)	0.81	0.368
	No		42 (32.3)	88 (67.7)		
Direct questions and exploration *	Yes	13 (14.3)	7 (53.8)	6 (46.2)	2.80	0.094
	No		38 (30.9)	85 (69.1)		
Others *	Yes	3 (3.3)	2 (66.7)	1 (33.3)	1.56	0.211
	No		43 (32.3)	90 (67.7)		

* Multiple response.

**Table 4:** Level of internalized stigma, social support, mental health literacy, depression, and stress (*N* = 136).

**Variables**	Range	Mean ± SD	Skewness	Kurtosis
**Internalized stigma**	1–4	2.20 ± 0.49	−0.05	0.36
**Social support**	1–5	3.25 ± 0.84	−0.27	0.10
**Mental health literacy**	1–5	3.15 ± 0.35	0.36	0.42
**Depression**	0–3	0.97 ± 0.64	0.61	−0.23
**Stress**	1–5	3.29 ± 0.94	−0.44	−0.04

**Table 5:** Factors affecting the utilization of community mental health services (*N* = 136).

Variables	OR (Odds Ratio)	95% CI (Confidence Interval)	*p*
Sex			
Men (ref. = Women)	3.33	1.17–9.48	0.025
Education (ref. = ≤High school)			
College	0.72	0.06–8.50	0.791
≥Master	0.37	0.03–4.36	0.432
Duration of illness (year)	2.31	1.23–4.32	0.009
Schizophrenia spectrum disorder			
Diagnosis (ref. = Non-diagnosis)	2.05	0.40–10.40	0.388
Bipolar disorder			
Diagnosis (ref. = Non-diagnosis)	1.09	0.19–6.10	0.924
Awareness of mental health services			
Recognition (ref. = Non-recognition)	39.09	2.40–637.23	0.010
Recognized through friends or family			
Yes (ref. = No)	1.37	0.46–4.02	0.571
Recognized through mental health professional			
Yes (ref. = No)	1.43	0.44–4.65	0.557
Internalized stigma	4.90	1.01–23.86	0.049
Depression	0.47	0.10–2.14	0.327
Stress	3.14	1.23–7.98	0.016

ref. = reference. Nagelkerke R^2^ = 0.60, Hosmer–Lemeshow X^2^ = 3.96 (*p* = 0.861).

## Data Availability

The raw data supporting the conclusions of this article will be made available by the authors upon reasonable request after signing a confidentiality agreement.
